# Learning from a crisis: a qualitative study on how nurses reshaped their work environment during the COVID-19 pandemic

**DOI:** 10.1186/s12912-024-02177-4

**Published:** 2024-07-29

**Authors:** Annemarie J. B. M. de Vos, Eline de Kok, Susanne M. Maassen, Monique Booy, Anne Marie J. W. M. Weggelaar-Jansen

**Affiliations:** 1 Academy of Nursing Science and Education, Elisabeth-TweeSteden Hospital, Hilvarenbeekse Weg 60, 5022 GC Tilburg, The Netherlands; 2 Fontys School of People and Health Studies, Fontys University of Applied Sciences, Professor Goossenslaan 1-01, 5022 DM Tilburg, The Netherlands; 3 Centre of Expertise Perspective in Health, Avans University of Applied Sciences, Hogeschoollaan 1, 4818 CR Breda, The Netherlands; 4 Nursing Staff Board, Amphia Hospital, Molengracht 21, 4818 CK Breda, The Netherlands; 5 Dutch Nurses’ Association, Orteliuslaan 1000, 3528 BD Utrecht, The Netherlands; 6 Julius Center for Health Sciences and Primary Care, University Medical Center Utrecht, Utrecht University, Universiteitsweg 100, 3584 CG Utrecht, The Netherlands; 7 Department of Quality and Patientcare, Erasmus University Medical Center Rotterdam, Dr. Molewaterplein 40, 3015 GD Rotterdam, The Netherlands; 8 Erasmus School of Health Policy and Management, Erasmus University Rotterdam, Burgemeester Oudlaan 50, 3062 PA Rotterdam, The Netherlands; 9 Tranzo, Tilburg University, Professor Cobbenhagenlaan 125, Tilburg, 5037 DB The Netherlands

**Keywords:** Autonomy, COVID-19, Nursing leadership, Nurse management, Qualitative research, Work environment

## Abstract

**Background:**

The global nursing shortages exacerbated by the COVID-19 pandemic necessitated a drastic reorganization in nursing practices. Work routines, the composition of teams and subsequently mundane nursing practices were all altered to sustain the accessibility and quality of care. These dramatic changes demanded a reshaping of the nurses’ work environment. The aim of this study was to explore how nurses reshaped their work environment in the early stages of the COVID-19 pandemic.

**Methods:**

A descriptive study comprising 26 semi-structured interviews conducted in a large Dutch teaching hospital between June and September 2020. Participants were nurses (including intensive care unit nurses), outpatient clinic assistants, nurse managers, and management (including one member of the Nurse Practice Council). The interviews were analysed with open, axial, and selective coding.

**Results:**

We identified five themes: 1) the Nursing Staff Deployment Plan created new micro-teams with complementary roles to meet the care needs of COVID-19 infected patients; 2) nurse-led adaptations effectively managed the increased workload, thereby ensuring the quality of care; 3) continuous professional development ensured adequate competence levels for all roles; 4) interprofessional collaboration resulted in experienced solidarity, a positive atmosphere, and increased autonomy for nurses; and, 5) supportive managers reduced nurses’ stress and improved work conditions.

**Conclusions:**

This study showed that nurses positively reshaped their work environment during the COVID-19 pandemic. They contributed to innovative solutions in an environment of equal interprofessional collaboration, which led to greater respect for their knowledge and competencies, enhanced their autonomy and improved management support.

**Supplementary Information:**

The online version contains supplementary material available at 10.1186/s12912-024-02177-4.

## Background

The nurses’ work environment is crucial to ensuring high-quality patient care [1, 2]. Damschroder et al. [3] define the work environment as the inner setting of the organization in which employees work. In a positive work environment nurses work autonomously, are in control of their practices, feel valued, respected and safe, and experience physical comfort, multidisciplinary collaboration, open communication, and career advancement [4]. Additionally, a favourable work environment is one where nurses are respected and valued based on their professionalism [5]. However, worldwide shortages of nurses have strained their work environment [6]. Nurses face high workloads, forced overtime, a lack of influence on their practices, an insufficient use of their competencies, and the sense of being unable to provide high-quality care [7, 8]. These important factors influence their job satisfaction and—combined with a less appealing work environment, low quality of work life, and poor treatment of management—are reasons for nurses to leave the profession.

The COVID-19 pandemic changed the nurses’ work environment drastically [9]. Nurses faced tremendous challenges to provide care, especially when the number of COVID-19-infected patients exceeded the available capacity, and access to hospital care for patients was strained. Modelling research shows an increase in workload, a 64% reduction of direct time for care, a 46% increase in time spent walking, and a 353% increase in missed care tasks per shift compared to pre-pandemic nursing care [10]. Nurses had to learn by experimenting how to care for patients with a new life-threatening disease for which no guidelines or standardized procedures were available [11–13]. Nurses also had to deal with the constant threat of infection (and thus infecting their family), the physical burden of working in protective clothing, and the constraint of social distancing in the workplace [9, 14–16]. Nurses were forced to be flexible as their roster continually changed, if colleagues became infected and were forced to isolate. Healthcare organizations had to make drastic changes to organizational structures to ensure proper care for COVID-19-infected patients. Consequently, nurses had to collaborate with new colleagues [17].

Although the negative effects of COVID-19 on nurses' health and wellbeing have been well reported [9, 18], less attention has been paid to how the nurses’ work environment changed and the way nurses responded to these changes. Chemali et al. [19] concluded from their systematic review that more research is needed to understand healthcare professionals’ experiences during the pandemic, as this ‘could contribute to building a sustainable health workforce and strengthening health systems for future pandemics’ [19].

Therefore, this study explored nurses’ experiences in undertaking the responsibility to reshape their work environment in response to the demands of the COVID-19 pandemic. We examined how nurses influenced the required measures, took responsibility for the quality of care, and altered their practices. Our research question was: how did nurses in a large Dutch teaching hospital reshape their work environment to address the changes needed during the early stages of the COVID-19 pandemic?

## Methods

### Design

We conducted a descriptive qualitative study [20] to capture various dimensions of the changes needed to address the challenges posed by the COVID-19 pandemic using semi-structured interviews to explore in-depth the reshaping of the nurses’ work environment [21]. We adhered to Standards for Reporting Qualitative Research (SRQR) [22], ensuring that all requirements were met [see Additional file 1].

### Setting

The study was conducted in a large Dutch teaching hospital (69,245 admissions and 93,221 outpatient visits per year; 4,385 employees in 2020). When the COVID-19 pandemic began in the Netherlands (March 2020), this hospital was one of the first to be flooded with COVID-19-infected patients. The hospital was forced to make decisions on capacity planning, operations management, and nurse deployment to meet the increasing demand for nursing care. Therefore, the hospital increased its intensive care unit (ICU) capacity from 22 to 46 beds and allocated three hospital floors (96 beds) to COVID-19 patients to better manage the continuous stream of critically ill patients admitted to the hospital. In March 2020, hospital policymakers developed the COVID-19 Nursing Staff Deployment Plan to provide nursing knowledge and skills, prevent capacity problems, and ensure sustainable nursing employment. This plan introduced three nursing level roles: A – final responsibility; B – executor; and C – support, each of which had specific tasks on the COVID-19 wards (Table 1). Nurses from other wards complemented the permanent ICU nursing staff and outpatient clinic assistants were assigned to the COVID-19 wards. Because of the rising number of COVID-19-infected patients, the plan was implemented in the second week of March 2020, when micro-teams of professionals in the three designated roles (A, B, and C) were formed. Nurses were temporarily transferred to the COVID-19 wards and expanded ICU, while other healthcare professionals were moved to other wards. To accommodate all the patients infected with COVID-19, other patients also had to be shifted.

**Table 1:** COVID-19 Nursing Staff Deployment Plan: roles, criteria, and tasks

**The A role** will be performed by a registered nurse (vocational or bachelor trained) who holds ultimate responsibility for all nursing care	
*Criteria*	Known on the particular ward
	Experienced nurse
	Able to function as a senior nurse
*Tasks:*	Coordinate nursing care (together with B role)
	Manage C role
	Attend ward rounds
	Administer medication
	Organize patient admission and discharge
**The B role** will be performed by a registered nurse (vocational or bachelor trained) who is responsible for providing nursing care to all patients	
*Criteria*	Preferably known on the particular ward
	Known to others in the hospital (e.g., psychiatry, geriatrics, paediatrics
	nurse practitioner, day treatment)
*Tasks*	Collaborate with A role
	Manage C role
	Provide nursing care to assigned patients
**The C role** will be performed by an outpatient clinic assistant or nursing student who is responsible for providing activities of daily living (ADL) care to patients with a low-complexity care needs	
*Criteria:*	Not known on the ward
	Accountable to A and B roles
*Tasks:*	Perform patient checks (e.g., Early Warning System score)
	Support patients with ADL
	Support patients with nutrition
	Assist A and B roles in complex care situations

### Participants

For the semi-structured interviews, we emailed invitations to all 245 nurses and outpatient clinic assistants (in the three roles classified in the Nursing Staff Deployment Plan). Only inclusion criterion was the participant had worked in one of the newly established COVID-19 wards during the first waves of the pandemic. We purposively invited another ten healthcare professionals via email who were either involved in developing and implementing the Nursing Staff Deployment Plan (*n* = 7) or were members of the Outbreak Management Team (*n* = 3) to gain more insight into hospital policy and the challenges faced. We included everyone who wanted or was able to participate.

In total 26 participants agreed to be interviewed (Table 2). One researcher (AdV) sent them an email outlining the study aim, confidentiality, data storage, and ethics. Subsequently the researcher planned face-to-face interviews at times that suited the participants. Although well-known in the hospital, this researcher was not involved in the management of the COVID-19 pandemic.

**Table 2:** Characteristics of participants (for more information on the Dutch context of nursing see Van Kraaij et al. [23]

n (*N* = 26)	Profession	Usual work setting	Work setting during COVID-19 pandemic	Additional roles and tasks during COVID-19 pandemic
1	ICU nurse	ICU/ED	COVID-19 ICU/ED	
7	ICU nurse	ICU	COVID-19 ICU	
8	Registered nurse	Hospital ward	COVID-19 ward	
1	Outpatient clinic assistant	Outpatient clinic	COVID-19 ward	
1	Nurse manager	ICU	COVID-19 ICU	OMT member
1	Nurse manager	Hospital ward	COVID-19 ward	
1	Nurse manager	Outpatient clinic	COVID-19 ICU	
1	Healthcare & operations manager	Management	Management	OMT member
1	Healthcare & operations manager	Management	Management	NSPD development and implementation
2	Healthcare & operations manager	Management	Management	OMT member, NSDP development and implementation
1	Member Nurse Practice Council	Nurse Practice Council	COVID-19 ICU	NSDP development and implementation
1	Member Hospital Board	Hospital Board	Hospital Board	OMT member

*ED* Emergency Department, *ICU* Intensive Care Unit, *NSDP* Nursing Staff Deployment Plan, *OMT* Outbreak Management Team

### Data collection and analysis

Two researchers (AdV and MB) developed a pre-defined topic list based on the Essentials of Magnetism [24, 25] [see Additional file 2]. This topic list was discussed with nurses to ensure the significance of the study. Aligning with Magnet principles enabled us to study relevant elements, including: 1) governance structures that empower nurses to participate in decision-making processes; 2) nurses’ personal leadership driving organizational change and innovation; 3) fostering a culture of nursing excellence; and 4) ultimately improving patient outcomes [26]. Between June and September 2020, semi-structured interviews (duration, 50 min on average) were conducted in person in the workplace. Audio-recordings of the interviews were transcribed verbatim, summarized, and anonymized by two researchers (AdV and MB) and accompanied by field notes. These notes described the setting, and the observations and thoughts of the researcher to reflect on and prevent bias and support memory recollection. The dependability and integrity of the transcripts were tested by a member check, which involved sending the transcripts to five participants. The member checks yielded no changes to the transcripts.

Next, two experienced researchers (AdV and EdK) began the data analysis by independently close reading each summary and undertaking multiple, reflexive thematic coding steps in inductive analysis [27, 28]. They compared the open codes of the first 15 interviews and discussed the code labels until they reached consensus. The same researchers then relabelled their first 15 transcripts and labelled the rest. In the next phase, one researcher (AdV) axial-coded the labels and then linked the codes in clusters. The whole research team, including the members not involved in either the hospital or data gathering, discussed until consensus was reached and situational findings were provided [29]. Next, the same researcher (AdV) performed selective coding to merge the clusters in themes. Ultimately, the entire research group identified and agreed upon the five themes described in the Results section. To ensure dependability, all research steps, including data collection, data analysis, and manuscript preparation were documented in a reflexive journal. Reflections, particularly potential preconceptions, were continuously crosschecked among the entire research team. Based on the results of our analysis we concluded that saturation had been achieved.

### Research team

The research team consisted of two female PhD-level researchers (AdV and AMWJ) with extensive experience in qualitative research methods, two female PhD students (EdK and SMM), and one female MSc-level researcher (MB) with substantial experience in qualitative research methods. One PhD-level researcher and the MSc-level researcher were affiliated with the study hospital during the study period; however, neither had any prior or ongoing connection with the participants.

## Results

Analysis of the qualitative data identified five themes related to the participants’ experience of the changes the Nursing Staff Deployment Plan caused to the work environment.

### The nursing staff deployment plan created new micro-teams with complementary roles

The Nursing Staff Deployment Plan described working with COVID-19-infected patients as a choice and called upon healthcare professionals’ willingness to do so:

*“It always is a voluntary choice whether or not to work on the COVID-19 ward. We cannot and must not force outpatient clinic assistants to do so. Every morning we had a meeting with all nurse managers to discuss the vacancies and deployment in the wards.” (Healthcare & operations manager)*

*“In my view, nurses had […] a say in whether they were willing to work on the COVID-19 ward, but I don’t know what happened in daily practice. I think nurses were quite encouraged by their managers to do it.” (Member hospital board)*

Most healthcare professionals were informed by their nurse manager via email about the different roles on the newly assigned ward. One manager said that the plan largely depended on the communication skills of the nurse managers, who knew their staff best and could explain the shifts in tasks and roles of the different teams to prevent stress and anxiety. However, most nurses expressed an intrinsic motivation to provide the needed care:

*“I chose this profession and these days you just have to do what is expected of you. If they expect me to work on the COVID-19 ward, then I should do just that. Because you just have to be there for the patients.” (Registered nurse)*

The Nursing Staff Deployment Plan reallocated nursing staff to the various roles of each new micro-team assigned to care for a specific number of COVID-19-infected patients.

*This meant working with a different mix of nursing expertise and that led to a lack of routine and competencies. Although we tried to limit this as much as possible, we didn’t always succeed.” (Healthcare & operations manager)*

### Nurse-led adaptations effectively managed the increased workload, thereby ensuring the quality of care

Participants reported that although more than enough staff were available on the COVID-19 wards, a surplus was necessary in case of a sudden influx of infected patients. This meant fewer nurses were available for other wards:

*“There were enough staff on the COVID-19 ward. Sometimes I saw lots of blue gowns [nurses] walking around. But we had a shortage on the other wards.” (Registered nurse)*

The Department of Process Innovation and the nurse managers determined the nurse-patient ratio needed to guarantee safe, high-quality care on the COVID-19 wards. The ratio was based on the anticipated complexity of care and the task division proposed in the Nursing Staff Deployment Plan. However, according to the participants, the nurse-patient ratio was based on the nurses’ experience, rather than evidence. Predicting the optimal nurse-patient ratio was difficult because the level of nursing care changed constantly, depending on the condition of COVID-19-infected patients, which could deteriorate quickly. Also, the size of micro-teams was not as consistent as intended because of staff changes due to illness, quarantine, or reallocation to other wards due to shortages. This undermined the agreed ratios:

*“I understood the justification for [creating] the A, B, and C roles, but daily practice was often different because of the unintended absence of the B or C role. Sometimes I felt responsible for ten patients, which was tough.” (Registered nurse)*

In response, nurses took the lead and balanced their capacity on each shift to ensure the best quality of care. They learned that the composition of the micro-team roles could be adjusted on an hourly basis, depending on the situation. This required both a flexible mindset and a good idea of healthcare staff capacity, to gain a real-time overview of staff surpluses and shortages. Nurses urged management to provide this overview:

*“So, I’m working on a plan. How are we going to arrange things better? We’d profit from a global overview of the plus and minus [of staffing], steering [capacity] centrally and not organizing it ward by ward.” (Healthcare & operations manager)*

### Continuous professional development ensured adequate competence levels for all roles

Participants agreed that up-to-date knowledge of COVID-19 care protocols was important for optimal nursing care. As one manager said: *“Knowledge is the professional’s strength. If you don’t know about certain aspects […] you can’t observe them.”* Because of the continuous stream of new evidence, even experienced ICU nurses could not rely on their previously acquired knowledge of ventilation management: *“Protocols change in the blink of an eye” (ICU nurse)*. Participants stressed that their knowledge of COVID-19 and how to provide the best care changed as they learned more about the virus:

*“I thought I knew enough, then I noticed that my knowledge was outdated, but that’s what happens with a new disease.” (Registered nurse)*

This quote shows how important and especially relevant the nurses’ experience-based knowledge was, given that the condition of COVID-19-infected patients could decline rapidly. Thus, new competencies and knowledge were needed to care for these patients. This prompted nurses to search the internet for the latest research and guidance on nursing and treating COVID-19-infected patients. Nurses took on the responsibility to acquire and teach the necessary knowledge to one another (on the job). For example, nurses from the respiratory ward gave instruction on the COVID-19 ward.

*“And then you start reading papers again. What’s going on? What are the newest insights? What are the current ventilation guidelines? What insights come from these guidelines? So, you’re constantly in the COVID-19 mode. And on top of that, you follow webinars and other things like that. […] You know nothing about it, and you want to give good care. [...] You have to be innovative and look for information in places where you normally wouldn’t look.” (ICU nurse)*

Nurses also called upon support departments to back their need for more knowledge. For example, staff from the Infection Prevention Department were invited to instruct healthcare staff on how to change into protective clothing. The Hospital Academy Department also offered novel e-learning opportunities and online videos on how to care for COVID-19 patients.

Moreover, nurses adapted routine practices with new guidelines, creating easier reporting templates, and developing new equipment/tooling:

*“We started packaging arterial blood gas material together in kits for quick and easy use, because we used them so many times a day*.”* (Outpatient clinic assistant)*

*“My colleagues created a ward-round chart and standard nursing report for our Electronic Patient Record, specifically for COVID-19 patients. Nobody expected that.” (Registered nurse)*

Despite this, participants indicated that some team members, especially outpatient clinic assistants and nursing students (both supportive C roles), were not competent enough to perform their assigned tasks. One nurse said:

*“Once a patient said he needed to go to the bathroom and the assistant [C role] thought he could just go. But I know that a patient with a 15-liter non-rebreathing mask can’t just walk to the toilet without the mask. Seems a simple thing. But, they [C role staff] should really ask. That didn’t happen, so things went wrong.” (Registered nurse)*

While some professionals in B and C roles told their colleagues in A roles that they did not always know what to do, others were reluctant to reveal their incompetence because of the hectic situation. Some were simply unaware of their incompetence: *“People don’t know what they don’t know” (nurse manager)*. In the beginning this had a negative impact on micro-team collaboration and patient care quality.

*“I found the new tasks with the three roles difficult. Some nurses and outpatient clinic assistants performed really well, but others thought it was scary. […] Sometimes I was busier instructing and reassuring them [C role staff] because I couldn’t do the half-hourly patient check-ups on my own.” (Registered nurse)*

This quote shows that working daily with colleagues with unknown competencies required A roles to have substantial communication and coaching skills:

*“Which tasks can this buddy [C role] do independently and which ones not? It depends on the individual. You need people who are open in their communication and clear about their boundaries.” (ICU nurse)*

To prevent miscommunication, avoidable incidents, and reduced quality of care, nurses created new routines for task division. For example, the A role instructed the C role to take the patients’ blood pressure and report back to the A role. They also organized daily learning and reflection sessions for micro-teams to establish trust in each other’s competencies.

In sum, the new task assignments in the micro-teams changed the work environment for all team members, sometimes daily, and this impacted the quality of patient care. We found that the success of a micro-team relied on competency enhancement for all, based on emerging evidence on COVID-19, on the job training by nurses’ and supporting department and development of tools. Additionally, the potential shortcomings of knowledge and skills of A-role members especially required open and respectful communication. Working in micro-teams included collaborating with other healthcare professionals and management, as the next two sections will show.

### Interprofessional collaboration resulted in experienced solidarity, a positive atmosphere, and increased autonomy for nurses

Not just nurses but physicians were also relieved of their normal duties to work in the new COVID-19 micro-teams.

*“One day I worked with a pathologist, who had to listen to the patient’s lungs. I think that he hadn’t seen a living patient for 20 years. Or the psychiatrist who came to assess a lung patient. […] I thought this is special, but I get it, because it’s an all-hands-on-deck crisis.” (Registered nurse)*

Participants described their new work environment as an increased interdisciplinary collaboration, characterized by frequent communication beyond the usual boundaries between disciplines. As mentioned above, healthcare staff were insecure about how to treat and care for COVID-19-infected patients because they lacked sufficient knowledge of the virus. Therefore, frequent interdisciplinary consultations were necessary to discuss different profession-based opinions to determine the best quality of care. Confronted by the COVID-19 crisis, many participants willingly collaborated and developed equal, mutually respectful, interdisciplinary relationships that they did not have before.

*“It was great! There were also plastic surgeons and gynaecologists. […] They said, ‘Let’s all do it together!’ I actually thought it was a very good atmosphere.” (Registered nurse)*

This quote illustrates the solidarity and positive atmosphere that our participants experienced. Some participants suggested that the [new] equality between professionals was fostered by the fact that everyone wore the same protective clothing. Others believed it was fostered by a shared feeling of despair caused by the COVID-19 pandemic.

*“Everyone helped each other. So, we all took turns turning ICU patients over, from their back onto their belly. Normally, physicians wouldn’t do this, but now everyone chipped in.” (Nurse manager)*

Nurses in particular found they had gained more professional autonomy than before. For example, when rapid intervention was needed and no physician was present, nurses had to make decisions independently. Also, the lack of scientific information on the virus meant that nurses became the hands-on experts, based on their experience of taking care of so many COVID-19-infected patients.

*“I decided to do some things on my own, but only if I could defend [my decision] properly. And I always discussed it with a doctor afterward. COVID-19 was a new disease for all of us. […] This contributed to the fact that the doctors respected our decisions and actions.” (Registered nurse)*

Nurses participated more in discussions on ward rounds and in multidisciplinary meetings to help enhance proactive treatment plans, including when to start palliative care. Nurses indicated that to their surprise physicians respected the nurses’ professional autonomy and valued their input on patient treatment.

The nurses’ professional autonomy extended to their making independent decisions on the tasks each role in the micro-team should perform. This required close collaboration with management, as we will show in the next section.

### Supportive managers reduced nurses’ stress and improved work conditions

Nurse managers had to manage the established micro-teams without being able to do team building or letting members become acquainted with each other first. Despite this, many participants reported a keen sense of belonging in their micro-teams. Nurses said that this feeling arose spontaneously upon taking on the new responsibilities. Once the crisis began, they just buckled down, worked without complaint, and took pride in their achievements:

*“Everyone wanted to help and tried to make the best of it. I felt a true team spirit with everyone regardless of function or discipline.” (Registered nurse)*

Nurses gained the support of their nurse managers by expressing their concerns and asking managers to advocate on their behalf. Participants emphasized the importance of their nurse managers being visible on the ward and available to share concerns. The managers’ ongoing support for their nurses’ mental wellbeing, relieved stress and increased their job satisfaction. For example, managers adjusted rosters so that staff could take short breaks, and ensured they had sufficient supplies of equipment, medication, and bandages.

*“I worked in a team that functioned like a well-oiled machine. I saw the nurse manager regularly, and she always asked how I was doing. If I needed anything or if any adjustments needed to be made. She emphasized the need to tell her these things, so the team could learn from the situation. They were very open to learning.” (Registered nurse)*

*“I’ve seen nurse managers standing up for their team. For example, when personal protective measures were not in place, they moved heaven and earth to get them.” (Member, Nurse Practice Council)*

Despite being preoccupied with deploying and rostering nursing staff, most nurse managers made time for a daily chat with individual team members during breaks, shift evaluations, or other meetings. Nurse managers also paid attention to the (ethical) concerns about patients and family that the nurses raised as this weighed emotionally on the nurses. Together with management, nurses organized (online) meetings to discuss their dilemmas, such as not being able to provide optimal care to patients or feeling obliged to come to work when they were afraid to do so.

*“Who else can do my job? That was the dilemma for lots of nurses. Actually, I don’t want to do it, but I just can’t refuse. […] Patients depend on my care, and if I don’t do my job, who would take care of these patients? I can’t just tell my nurse manager that I’m not coming to work because I find it scary and I’m afraid, or because I have a vulnerable parent.” (Registered nurse)*

This shows that, even if participants were stressed by their working conditions, they had secured the support of nurse managers on the emotional level and in work routines.

## Discussion

This study explored how nurses reshaped their work environment during the COVID-19 pandemic. We revealed how nurses coped with changes in tasks and team composition in the early stages of the pandemic. Without any knowledge of this new disease, professionals from different backgrounds with different competencies worked together in newly formed micro-teams to care for COVID-19-infected patients. Nurses coordinated the assignment of work to the three roles (A, B, and C) and began innovating care processes to safeguard the quality of care. The nurses in our study found new ways to cope with existing rules and regulations and new ways to reshape their work environment. Other studies have also demonstrated how nurses all over the world were forced to continuously reshape their working conditions and alter their working routines as new information about the virus became available [11, 12, 17, 30].

Our findings illustrate how the rigorous changes in the nurses’ working conditions – on top of existing shortages and work environment issues [6, 7] – could have reduced job satisfaction and increased the nursing staff attrition reported by many other scholars [31, 32]. However, our study shows that effective communication and collaboration created a positive work environment even during the dire circumstances of the COVID-19 pandemic [33] that enhanced the value of nurses in the eyes of other professionals and management. Other studies also show that nurses, who form the largest proportion of healthcare professionals, played a key role in responding to the crisis [34]. Their hard work became visible and was respected by the public too [35, 36].

Similar to our study, Wei et al. [37] show that exhibiting leadership in daily practice is linked to a positive work environment. The COVID-19 pandemic motivated nurses to show leadership to reshape their work environment and thereby cope with the circumstances [38]. Our study demonstrates that reshaping the nurses’ working environment increased their professional autonomy in both decision-making and delivering good quality patient care. For example, nurses took the initiative to educate themselves and their team members about the virus and the skills needed to take care of patients. Investigating the changing work environment in the early stages of the COVID-19 pandemic, Thull-Freedman et al. [39] also observe the relevance of the expert knowledge and decision-making among frontline staff.

Furthermore, our study shows nurses developing equal interprofessional collaborations which allowed them to express their opinion on the best care for a patient. This became part of their increasing professional autonomy and professionalization. Nurses took control of their own practices and assessed and decided independently whether a situation was urgent. Miawati et al. [36] also found that nurses were forced to be creative and innovative and adapt their existing behaviour, norms and standards and thus gained room to reshape patient care. Nurses sharing their expertise with physicians increased their sense of collaboration, equality, acceptance, and equivalence. The literature shows that good communication and constructive collaboration improve the quality of patient care, because decision-making is more effective when everyone’s view is taken into account [40]. Additionally, several studies show how effective communication, teamwork, and personal leadership create a positive work environment, which in turn can enhance nursing staff retention [24, 41, 42].

We found that when nurses voiced their concerns, nurse managers could provide support by advocating on their behalf and safeguarding the supply chain for equipment. Furthermore, managers supported nurses by discussing their ethical concerns and allowing them to show leadership. Their support contributed to reducing the physical workload and mental stress of nurses, as other researchers have also reported [39, 43], providing evidence that a nurse manager’s leadership style positively affects the work environment of frontline nurses [44, 45].

This qualitative study has some limitations. Firstly, the findings represent the opinions and insights of a (purposefully) sampled group from only one Dutch hospital. Outpatient clinic assistants and nursing students (the C role) are particularly underrepresented. Moreover, as only 10% of potential participants were selected for inclusion, it could be possible that a group with specific opinions or feelings agreed to an interview. This selection has a potential impact on the findings so caution is advised when interpreting our results. Future studies should include a larger sample size and interview participants from several hospitals (in different countries) to better understand how nurses reshape their work environment and show leadership in response to new (crisis) circumstances. However, given the qualitative design of our study, we were able to fulfil our aim of providing a rich description of the collected data and capturing the various aspects of the unprecedented situation in 2020.

Secondly, our participants' views on the nurses’ work environment may have been affected by their elevated levels of stress and sense of relief just after the first wave of the pandemic. Participants might have expressed different opinions and feelings on the situation over time, after the second and following waves. More long-term research is needed to examine the effect of time on participants’ opinions. Additionally, the study could have benefited from data triangulation, for example with observations. However, this was not possible due to the COVID-19 pandemic restrictions.

Thirdly, the interviewers (AdV and MB) worked in the hospital during the period of data collection. This proximity enhanced our participants sensitivity to relevant issues but it may have caused bias. However, both interviewers have extensive experience in interviewing and the entire research team tested the credibility of the findings by carefully discussing the major themes and key points of all interviews. To maximize dependability, all steps – from data collection and analysis through to manuscript preparation – were well-documented and assessed by the entire team, including the researchers working outside the hospital (AMWJ, EdK and SMM).

## Conclusions

The COVID-19 pandemic substantially affected the nurses’ work environment. Our study shows that the Nursing Staff Deployment Plan required substantial changes to daily routines as the nurses’ usual teams were combined and supplemented by other personnel (outpatient clinic assistants and nursing students). Nurses responded to these circumstances by: 1) exhibiting leadership in their micro-teams; 2) educating of their colleagues; and 3) innovating care processes. With this response, nurses shaped their work environment so that they could perform their job well under the harsh pandemic conditions. The nurses ensured that they gained their managers’ support, not just for the new work routines but by facilitating sufficient equipment and advocating on their behalf. Furthermore, interprofessional collaboration increased, especially with physicians, which resulted in a more positive work environment for nurses, as they gained more respect and more professional autonomy due to their experience-based knowledge.

### Implications for nursing management

The COVID-19 pandemic changed several crucial aspects of the working environment of nurses. Our study reveals that nurses were able to positively influence their work environment. We offer insights into nurses showing leadership in times of crisis from organizational and cultural perspectives. In crisis situations, when existing rules, regulations, policies, and knowledge shifted or were no longer applicable, nurses took responsibility for their own and other professionals’ professionalization. They created innovative solutions in close and equal interprofessional collaboration and increased their autonomy.

This study reminds nurse managers of the importance of their influential supportive position, backing up nurses who are exhibiting leadership to positively reshape their work environment. Our findings suggest that nurse managers should collaborate further with nurses to develop strategies that stimulate leadership in nurses, thus permitting nurses to take a leading role in reshaping their own work environment. This is particularly important given the shortages of nurses worldwide and the need for nursing managers to prepare for a future pandemic.

### Supplementary Information

Supplementary Material 1.

Supplementary Material 2.

## Data Availability

The data that support the findings of this study are not openly available due to reasons of sensitivity and are available from the corresponding author upon reasonable request.
