# Supplementary material for: Learning from a crisis: a qualitative study on how nurses reshaped their work environment during the COVID-19 pandemic
Source: BMC Nurs. 2024 Jul 29;23:515. doi: 10.1186/s12912-024-02177-4 (PMC11287935; doi:10.1186/s12912-024-02177-4)
Supplement: Supplementary file 1 — Supplementary Material 1. [file 12912_2024_2177_MOESM1_ESM.docx]

**Standards for Reporting Qualitative Research (SRQR)**

|  | **Title and abstract** |  |  |
| --- | --- | --- | --- |
| S1 | Title | Concise description of the nature and topic of the study Identifying the study as qualitative or indicating the approach (e.g., ethnography, grounded theory) or data collection methods (e.g., interview, focus group) is recommended | *Learning from a crisis: a qualitative study on how nurses reshaped their work environment.* |
| S2 | Abstract | Summary of key elements of the study using the abstract format of the intended publication; typically includes background, purpose, methods, results, and conclusions | *Conform requirements of the journal* |
|  | **Introduction** |  |  |
| S3 | Problem formulation | Description and significance of the problem/phenomenon studied; review of relevant theory and empirical work; problem statement | *Worldwide shortages of nurses have strained their work environment. Factors, such as high workloads, forced overtime and a lack of influence on their practices influence nurses’ job satisfaction and contribute to nurses leaving the profession.*  *Due to the COVID-19 pandemic, nurses’ work environment changed drastically. As a consequence, they had to learn by experimentation how to care for patients with a new life-threatening disease and to deal with the constant threat of infection, the physical burden of working in protective clothing, and the constraint of social distancing. Thus far, there is limited understanding regarding healthcare professionals’ experiences in taking responsibility to reshape the work environment in response to the demands of the COVID-19 pandemic.* |
| S4 | Purpose or research question | Purpose of the study and specific objectives or questions | *This study explored nurses’ experiences in undertaking the responsibility to reshape their work environment in response to the demands of the COVID-19 pandemic. We examined how nurses influenced the required measures, took responsibility for the quality of care, and altered their practices.*  *How did nurses in a large Dutch teaching hospital reshape their work environment to address the changes needed during the early stages of the COVID-19 pandemic?* |
|  | **Methods** |  |  |
| S5 | Qualitative approach and research paradigm | Qualitative approach (e.g., ethnography, grounded theory, case study, phenomenology, narrative research) and guiding theory if appropriate; identifying the research paradigm (e.g., postpositivist, constructivist/ interpretivist) is also recommended; rationale | *We conducted a descriptive qualitative study, using semi-structured interviews to explore in-depth the reshaping of the nurses’ work environment. Next, we undertook reflexive thematic inductive analysis.* |
| S6 | Researcher characteristics and reflexivity | Researchers’ characteristics that may influence the research, including personal attributes, qualifications/experience, relationship with participants, assumptions, and/or presuppositions; potential or actual interaction between researchers’ characteristics and the research questions, approach, methods, results, and/or transferability | *The interviewers worked in the hospital during the period of data collection. This helped our participants to be sensitive to relevant issues but it may have caused bias. However, both interviewers have extensive experience in interviewing and the entire research team tested the credibility of the findings by carefully discussing the major themes and key points of all interviews.* |
| S7 | Context | Setting/site and salient contextual factors; rationale | *The study was conducted in a large Dutch teaching hospital (69,245 admissions and 93,221 outpatient visits per year; 4,385 employees in 2020). When the COVID-19 pandemic began in the Netherlands (March 2020), this hospital was one of the first to be flooded with COVID-19-infected patients.* |
| S8 | Sampling strategy | How and why research participants, documents, or events were selected; criteria for deciding when no further sampling was necessary (e.g., sampling saturation); rationale | *For the semi-structured interviews, we invited all 245 nurses and outpatient clinic assistants who had worked in one of the newly established COVID-19 wards during the first waves. Additionally, we purposively invited ten healthcare professionals who were either involved in developing and implementing the Nursing Staff Deployment Plan (n=7) or were members of the Hospital Outbreak Management Team (n=3) to gain more insight into hospital policy and the challenges faced. We included everyone who wanted or was able to participate.*  *In total 26 participants expressed willingness to be interviewed and were contacted by one researcher via an email outlining the study aim, confidentiality, data storage, and ethics.*  *Based on the results of the data analysis we concluded that saturation had been achieved.* |
| S9 | Ethical issues pertaining to human subjects | Documentation of approval by an appropriate ethics review board and participant consent, or explanation for lack thereof; other confidentiality and data security issues | *All participants were informed of the study objectives and provided written informed consent before their semi-structured interview. Data were collected and stored in line with the Dutch General Data Protection Regulation. The study was approved by [blinded for review].* |
| S10 | Data collection methods | Types of data collected; details of data collection procedures including (as appropriate) start and stop dates of data collection and analysis, iterative process, triangulation of sources/methods, and modification of procedures in response to evolving study findings; rationale | *We conducted a descriptive qualitative study to capture various dimensions of the changes needed to address the challenges posed by the COVID-19 pandemic using semi-structured interviews to explore in-depth the reshaping of the nurses’ work environment…. For the semi-structured interviews, we invited all 245 nurses and outpatient clinic assistants (in the three roles classified in the Nursing Staff Deployment Plan) who had worked in one of the newly established COVID-19 wards during the first waves of the pandemic. We purposively invited another ten healthcare professionals who were either involved in developing and implementing the Nursing Staff Deployment Plan (n=7) or were members of the Hospital Outbreak Management Team (n=3) to gain more insight into hospital policy and the challenges faced. We included everyone who wanted or was able to participate.* |
| S11 | Data collection instruments and technologies | Description of instruments (e.g., interview guides, questionnaires) and devices (e.g., audio recorders) used for data collection; if/how the instrument(s) changed over the course of the study | *Two researchers developed a pre-defined topic list based on the Essentials of Magnetism (Kramer et al., 2008; Mcclure et al., 1983) (see Appendix 1). This topic list was discussed with nurses, to ensure the relevance of the study. By aligning with Magnet principles, we were able to study elements relevant for nurses’ work environment, including 1) governance structures that empower nurses to participate in decision-making processes, 2) driving organizational change and innovation through personal leadership, 3) fostering a culture of nursing excellence, and 4) ultimately improving patient outcomes. Next, face to face semi-structured interviews were conducted between June and September 2020, at the workplace, which lasted 50 minutes on average. The audio recordings of the interviews were transcribed verbatim, summarized, and anonymized by two researchers and accompanied by field notes. The field notes described the setting, and the observations and thoughts of the researcher to reflect and prevent bias and support memory recollection. The reliability and integrity of the transcripts were tested by a member check, which involved sending the transcripts to five participants. The member checks yielded no changes to the transcripts*. |
| S12 | Units of study | Number and relevant characteristics of participants, documents, or events included in the study; level of participation (could be reported in results) | *We invited all 245 nurses and outpatient clinic assistants who had worked in one of the newly established COVID-19 wards during the first waves. Additionally, we purposively invited ten healthcare professionals who were either involved in developing and implementing the Nursing Staff Deployment Plan (n=7) or were members of the Hospital Outbreak Management Team (n=3) to gain more insight into hospital policy and the challenges faced. We included everyone who wanted or was able to participate.*  *In total 26 participants were included in the study. Characteristics, such as profession, usual work setting, work setting during COVID-19 pandemic and additional roles and tasks during COVID-19 pandemic are presented in Table 1.* |
| S13 | Dataprocessing | Methods for processing data prior to and during analysis, including transcription, data entry, data management and security, verification of data integrity, data coding, and anonymization/deidentification of excerpts | *The audio recordings of the interviews were transcribed verbatim, summarized, and anonymized by two researchers and accompanied by field notes. The field notes described the setting, and the observations and thoughts of the researcher to reflect and prevent bias and support memory recollection.* |
| S14 | Data analysis | Process by which inferences, themes, etc., were identified and developed, including the researchers involved in data analysis; usually references a specific paradigm or approach; rationale | *Two experienced researchers began the data analysis by independently close reading each summary and undertaking multiple reflexive thematic inductive analysis coding steps (Gehman et al., 2018; Williams & Moser, 2019). They compared the open codes of the first 15 interviews and discussed the code labels until consensus was reached. The same researchers then relabelled their first 15 transcripts as well as labelled the rest. In the next phase, one researcher axial-coded the labels and then linked the codes in clusters. The whole research team, including members not involved in the hospital nor data gathering, discussed until consensus was reached and situational findings were provided (Bowen, 2006). Next, the same researcher performed selective coding to merge the clusters in themes. Ultimately, the whole research group identified and agreed upon the five themes described in the Results section.* |
| S15 | Techniques to enhance trustworthiness | Techniques to enhance trustworthiness and credibility of data analysis (e.g., member checking, audit trail, triangulation); rationale | *The reliability and integrity of the transcripts were tested by a member check, which involved sending the transcripts to five participants. The member checks yielded no changes to the transcripts.*  *To ensure dependability, all research steps, including data collection, data analysis, and manuscript preparation, were documented in a reflexive journal. Reflections, particularly potential preconceptions, were continuously crosschecked among the entire research team.* |
|  | **Results/ findings** |  |  |
| S16 | Synthesis and interpretation | Main findings (e.g., interpretations, inferences, and themes); might include development of a theory or model, or integration with prior research or theory | *We identified five themes: 1) the Nursing Staff Deployment Plan created new micro-teams with complementary roles to meet the care needs of COVID-19 infected patients; 2) nurse-led adaptations effectively managed the increased workload, thereby ensuring the quality of care; 3) continuous professional development activities ensured adequate competence levels for all roles; 4) interprofessional collaboration resulted in experienced solidarity, a positive atmosphere, and increased autonomy for nurses; and, 5) nurse managers supported nurses resulting in reduced stress and improved work conditions.* |
| S17 | Links to empirical data | Evidence (e.g., quotes, field notes, text excerpts, photographs) to substantiate analytic findings | *Throughout the manuscript we have used 22 quotes to substantiate the findings. E.g. “I worked in a team that functioned like a well-oiled machine. I saw the nurse manager regularly, and she always asked how I was doing. If I needed anything or if any adjustments needed to be made. She emphasized the need to tell her these things, so the team could learn from the situation. They were very open to learning.” (Registered nurse)* |
|  | **Discussion** |  |  |
| S18 | Integration with prior work, implications, transferability, and contribution(s) to the field | Short summary of main findings; explanation of how findings and conclusions connect to, support, elaborate on, or challenge conclusions of earlier scholarship; discussion of scope of application/ generalizability; identification of unique contribution(s) to scholarship in a discipline or field | *This study explored how nurses reshaped their work environment during the COVID-19 pandemic. We revealed how nurses coped with changes in tasks and team composition in the early stages of the pandemic. Without any knowledge of this new disease, professionals from different backgrounds with different competencies worked together in newly formed micro-teams to care for COVID-19-infected patients. Nurses coordinated the assignment of work to the three roles (A, B, and C) and began innovating care processes to safeguard the quality of care. The nurses in our study found new ways to cope with existing rules and regulations and new ways to reshape their work environment. Other studies have also demonstrated how nurses all over the world were forced to continuously reshape their working conditions and alter their working routines as new information about the virus became available (Eysenbach, 2020; Gómez-Ochoa et al., 2021; Islam et al., 2020; Mira et al., 2020).* *(Eysenbach, 2020; Gómez-Ochoa et al., 2021; Islam et al., 2020; Mira et al., 2020). Our findings illustrate how the rigorous changes in the nurses’ working conditions – on top of existing shortages and work environment issues (Burmeister et al., 2019; Harris, 2019; World Health Organization, 2020) – could have reduced job satisfaction and increased the nursing staff attrition reported by many other scholars (e.g., Brown et al., 2018; Cicolini et al., 2014; Gellasch, 2015; Özgür & Tektaş, 2018). However, our study shows that effective communication and collaboration created a positive work environment also during the dire circumstances of the COVID-19 pandemic that enhanced the value of nurses in the eyes of other professionals and management. Other studies also show that nurses, who form the largest proportion of healthcare professionals, played a key role in responding to the crisis. Their hard work became visible and was respected by the public too (e.g., Aquila et al., 2020; Miawati et al., 2021).* |
| S19 | Limitations | Trustworthiness and limitations of findings | *This study has some limitations. Firstly, the findings represent the opinions and insights of a (purposefully) sampled group from only one Dutch hospital. Outpatient clinic assistants and nursing students (the C role) are particularly underrepresented. Moreover, only 10% of potential participants were selected for inclusion; it could be possible that a group with specific opinions or feelings agreed to an interview. Therefore, this potentially impacted the findings, and consequently, caution is advised when interpreting our results.*  *Secondly, our participants' views of on the nurses’ work environment might have been affected by their elevated levels of stress and relief just after the first wave of the pandemic. Participants might have expressed different opinions and feelings on the situation over time, after the second or following waves.*  *Thirdly, the interviewers worked in the hospital during the period of data collection. This helped our participants to be sensitive to relevant issues but it may have caused bias. However, both interviewers have extensive experience in interviewing and the entire research team tested the credibility of the findings by carefully discussing the major themes and key points of all interviews. To maximize reliability, all steps – from data collection and analysis to manuscript preparation – were well-documented and assessed by the whole research team, including researchers working outside the hospital.* |
| S20 | Conflicts of interest | Potential sources of influence or perceived influence on study conduct and conclusions; how these were managed | *The researchers have no conflicts of interest.* |
| S21 | Funding | Sources of funding and other support; role of funders in data collection, interpretation, and reporting | *The researchers received no specific funding for this work.* |
