# Supplementary material for: Learning from a crisis: a qualitative study on how nurses reshaped their work environment during the COVID-19 pandemic
Source: BMC Nurs. 2024 Jul 29;23:515. doi: 10.1186/s12912-024-02177-4 (PMC11287935; doi:10.1186/s12912-024-02177-4)
Supplement: Supplementary file 2 — Supplementary Material 2. [file 12912_2024_2177_MOESM2_ESM.docx]

**Topic list for the semi-structured Interviews**

| Literature synthesis on Essentials of Magnetism (adapted from de Brouwer (2019)) | |
| --- | --- |
| Clinically competent peers | Nurses consider working with clinically competent fellow nurses important for delivering quality care. They regard specialty certification, educational degree, and both formal and informal peer review and reinforcement as proof of clinical competency. The absence of clinically competent peers inhibits quality patient care and is disadvantageous for nurse job satisfaction (Aiken et al., 2017; Kramer & Schmalenberg, 2004; Stalpers et al., 2017). |
| Collaborative nurse-physician relationships | In order to be collaborative, nurses and physicians should work together with mutual respect, trust, and equal power. Collaborative and collegial nurse-physician relationships increase retention of nurses and lower stress levels among nurses. More importantly, patients benefit from those relationships (Klipfel et al., 2014; Kramer & Schmalenberg, 2004; Tang et al., 2013). |
| Clinical autonomy | Clinical autonomy is defined as the freedom to independently make informed decisions that exceed standard nursing practice in the best interest of the patient (Kramer & Schmalenberg, 2008). Accountability of nurses in a positive and constructive manner is seen as an important element of the nursing work environment to enable high-quality patient care (Mensik, 2007). Nurses’ job satisfaction, levels of burnout, intention to leave the organization, as well as teamwork have been linked to nurses’ autonomy (Rafferty et al., 2001). |
| Nurse manager support | Attracting and retaining nurses, and nurse job satisfaction are affected by the support of nurse managers. Support means that nurse managers meet the expectations of nurses and provide them with means to deliver their job professionally, while also meeting the expectations of their superiors. Strong leadership is an important driver of adequate staffing, collaborative interdisciplinary relationships, and nursing participation in governance and policy development which positively affect nurse-sensitive outcomes (Goedhart et al., 2017; Laschinger & Leiter, 2006). |
| Control over nursing practice | Control over nursing practice is a democratic process facilitated by a visible, organized, and supportive structure. The structure should give nurses input and involvement in decision-making concerning clinical policies and problems and personnel issues which have an effect on nurses. Control over nursing practice, for instance in the form of a Nurse Practice Council, will only lead to the desired outcomes if nurses have the authority to take control over their daily practice (Laschinger & Wong, 1999). |
| Support for education | Education includes continuing education and short courses, as well as on- and offsite degree programs. Educational support is valued highly with a view to attracting and retaining nurses, quality patient care, and job satisfaction (Aiken et al., 2013; Schmalenberg & Kramer, 2008; Stalpers et al., 2017). Support for education is considered essential for the autonomous practice of nurses and for positive nurse-physician relationships. |
| Adequacy of staffing | Adequate nurse staffing is associated with lower mortality rates in hospitals in the United States, European and other countries (Aiken et al., 2014; Griffiths et al., 2016; Twigg et al., 2010). Staffing adequacy involves the number of nurses on a ward as well as the ability to deliver quality patient care. Nursing care left undone due to a lack of time is related to insufficient nurse staffing (Ausserhofer et al., 2014; Ball et al., 2014), mortality following common surgical procedures (Ball et al., 2018), lower nurse-perceived patient safety (Ball et al., 2014). |
| Patient-centered cultural values | Organizational culture can be defined as a patterned, shared system of values guiding behavior in the work setting (Kramer et al., 2008). Shared values and norms are the two elements that compose an organizations’ culture. Patient-centered culture is an important element of the nursing practice environment to enable high-quality patient care delivery (Kramer et al., 2008; Stalpers et al., 2017). |
| References  Aiken, L. H., Sloane, D., Griffiths, P., Rafferty, A. M., Bruyneel, L., McHugh, M., Maier, C. B., Moreno-Casbas, T., Ball, J. E., Ausserhofer, D., & Sermeus, W. (2017). Nursing skill mix in European hospitals: cross-sectional study of the association with mortality, patient ratings, and quality of care. BMJ Quality & Safety, 26(7), 559–568. https://doi.org/10.1136/BMJQS-2016-005567  Aiken, L. H., Sloane, D. M., Bruyneel, L., van den Heede, K., Griffiths, P., Busse, R., Diomidous, M., Kinnunen, J., Kózka, M., Lesaffre, E., McHugh, M. D., Moreno-Casbas, M. T., Rafferty, A. M., Schwendimann, R., Scott, P. A., Tishelman, C., Van Achterberg, T., Sermeus, W. (2014). Nurse staffing and education and hospital mortality in nine European countries: A retrospective observational study. The Lancet, 383(9931), 1824–1830. https://doi.org/10.1016/S0140-6736(13)62631-8  Aiken, L. H., Sloane, D. M., Bruyneel, L., van den Heede, K., & Sermeus, W. (2013). Nurses’ reports of working conditions and hospital quality of care in 12 countries in Europe. International Journal of Nursing Studies, 50(2), 143–153. https://doi.org/10.1016/J.IJNURSTU.2012.11.009  Ausserhofer, D., Zander, B., Busse, R., Schubert, M., de Geest, S., Rafferty, A. M., Ball, J., Scott, A., Kinnunen, J., Heinen, M., Sjetne, I. S., Moreno-Casbas, T., Kózka, M., Lindqvist, R., Diomidous, M., Bruyneel, L., Sermeus, W., Aiken, L., & Schwendimann, R. (2014). Prevalence, patterns and predictors of nursing care left undone in European hospitals: results from the multicountry cross-sectional RN4CAST study. BMJ Quality & Safety, 23(2), 126–135. https://doi.org/10.1136/BMJQS-2013-002318  Ball, J. E., Bruyneel, L., Aiken, L. H., Sermeus, W., Sloane, D. M., Rafferty, A. M., Lindqvist, R., Tishelman, C., & Griffiths, P. (2018). Post-operative mortality, missed care and nurse staffing in nine countries: A cross-sectional study. International Journal of Nursing Studies, 78, 10–15. https://doi.org/10.1016/J.IJNURSTU.2017.08.004  Ball, J. E., Murrells, T., Rafferty, A. M., Morrow, E., & Griffiths, P. (2014). Care left undone during nursing shifts: Associations with workload and perceived quality of care. BMJ Quality & Safety, 23(2), 116–125. https://doi.org/10.1136/bmjqs-2012-001767  de Brouwer, B. J. M. (2019). Essential elements of an excellent nursing practice. Radboud University Nijmegen. https://accuralis.com/wp-content/uploads/2021/06/Proefschrift-Essential-elements-of-an-excellent-nursing-practice-environment-Brigitte-de-Brouwer.pdf  Goedhart, N. S., van Oostveen, C. J., & Vermeulen, H. (2017). The effect of structural empowerment of nurses on quality outcomes in hospitals: a scoping review. Journal of Nursing Management, 25(3), 194–206. https://doi.org/10.1111/JONM.12455  Griffiths, P., Ball, J., Murrells, T., Jones, S., & Rafferty, A. M. (2016). Registered nurse, healthcare support worker, medical staffing levels and mortality in English hospital trusts: a cross-sectional study. BMJ Open, 6(2), e008751. https://doi.org/10.1136/BMJOPEN-2015-008751  Klipfel, J. M., Carolan, B. J., Brytowski, N., Mitchell, C. A., Gettman, M. T., & Jacobson, T. M. (2014). Patient safety improvement through in situ simulation interdisciplinary team training. Urologic Nursing, 34(1), 39–46. https://doi.org/10.7257/1053-816X.2014.34.1.39  Kramer, M., & Schmalenberg, C. (2004). Development and Evaluation of Essentials of Magnetism Tool. Journal of Nursing Administration, 34(7), 365–378. https://doi.org/10.1097/00005110-200407000-00010  Kramer, M., & Schmalenberg, C. (2008). Confirmation of a Healthy Work Environment. Critical Care Nurse, 28(2), 56–63. https://doi.org/10.4037/CCN2008.28.2.56  Kramer, M., Schmalenberg, C., & Maguire, P. (2008). Essentials of a magnetic work environment. Nursing, 23–27. https://doi.org/10.1097/01.NURSE.0000304729.97476.61  Laschinger, H. K., & Leiter, M. P. (2006). The impact of nursing work environments on patient safety outcomes: the mediating role of burnout/engagement. The Journal of Nursing Administration, 36(5), 259–267. https://doi.org/10.1097/00005110-200605000-00019  Laschinger, H. K., & Wong, C. (1999). Staff nurse empowerment and collective accountability: effect on perceived productivity and self-rated work effectiveness. Nursing Economics, 17(6), 308–316, 351. https://europepmc.org/article/med/10711183  Mensik, J. S. (2007). The essentials of magnetism for home health. Journal of Nursing Administration, 37(5), 230–234. https://doi.org/10.1097/01.NNA.0000269742.40137.A6  Rafferty, A. M., Ball, J., & Aiken, L. H. (2001). Are teamwork and professional autonomy compatible, and do they result in improved hospital care? Quality in Health Care , 10(Suppl II), ii32-ii37. https://doi.org/10.1136/qhc.0100032..  Schmalenberg, C., & Kramer, M. (2008). Essentials of a productive nurse work environment. Nursing Research, 57(1), 2–13. https://doi.org/10.1097/01.NNR.0000280657.04008.2a  Stalpers, D., Van Der Linden, D., Kaljouw, M. J., & Schuurmans, M. J. (2017). Nurse-perceived quality of care in intensive care units and associations with work environment characteristics: a multicentre survey study. Journal of Advanced Nursing, 73(6), 1482–1490. https://doi.org/10.1111/JAN.13242  Tang, C. J., Chan, S. W., Zhou, W. T., & Liaw, S. Y. (2013). Collaboration between hospital physicians and nurses: An integrated literature review. International Nursing Review, 60(3), 291–302. https://doi.org/10.1111/INR.12034  Twigg, D., Duffield, C., Thompson, P. L., & Rapley, P. (2010). The impact of nurses on patient morbidity and mortality – the need for a policy change in response to the nursing shortage. Australian Health Review, 34(3), 312–316. https://doi.org/10.1071/AH08668 | |
